# Social, psychological, and environmental-structural factors determine consistent condom use among rural-to-urban migrant female sex workers in Shanghai China

**DOI:** 10.1186/1471-2458-12-599

**Published:** 2012-08-03

**Authors:** Xiuxia Ye, Meili Shang, Tian Shen, Bei Pei, Xueqin Jiang, Yong Cai

**Affiliations:** 1 Shanghai Children’s Medical Center affiliated to Shanghai Jiao Tong University School of Medicine, Shanghai Key Laboratory of Children’s Environmental Health, No.1678, Dongfang Road, Shanghai, 200127, PR China; 2 Sanlin Community Sanitary Service Center, Pudong New Area, No. 375, Sanlin Road, Shanghai, 200126, PR China; 3 School of Public Health affiliated with Shanghai Jiaotong University School of Medicine, No.227, South Chongqing Road, Shanghai, 200025, PR China

## Abstract

**Background:**

To determine potential social, psychological, and environmental-structural factors that may result in motivating female sex workers (FSWs), who are rural-to-urban migrants, and their paying partners in Shanghai, China to promote consistent condom use (CCU).

**Methods:**

A cross-sectional study was conducted in five districts of Shanghai, including three suburbs and two downtown locales. We adopted a cluster randomized sampling method to obtain 20 geographic sites, which consisted of 1 or more communities/villages proximal to a location where FSWs were accessible. Five hundred four FSWs from 132 Xitou Fang (shampoo wash rooms), massage parlors, and hair salons who explicitly provided sexual services were enrolled in the study. Each participant completed a questionnaire survey and interview aimed to collect information on the perceptions and behaviors of individuals associated with a risk for human immunodeficiency virus/acquired immunodeficiency syndrome(HIV/AIDS),self-efficacy at negotiating safe sex,and the physical, social, and policy environment of the establishments where they worked.

**Results:**

The percentage of FSWs who reported consistent condom use with their paying partners was 63.3%. Controlling for socio-demographic characteristics in multivariate analyses, environmental-structural support (OR, 3.96; CI, 2.52–6.22) for condom use was the most significant positive predictor of CCU among FSWs and their regular paying partners. A high perception of susceptibility and risk of HIV/AIDS (OR, 1.96; CI, 1.25–3.01), a high perception of benefits on condom use to protect themselves (OR, 2.06; CI, 1.32–3.22), and high safe sex self-efficacy (OR, 2.52; CI, 1.64–3.85) also play important roles on CCU based on multivariate analyses.

**Conclusions:**

Environmental-structural factor support for condom use, in addition to social, psychological, and individual cognitive factors are significant predictors of CCU among FSWs, which should be assessed and addressed in research and interventions related to HIV/AIDS prevention among FSWs in China.

## Background

China is the largest low or middle income nation, and carries a large amount of rural-to-urban migrants (> 200 million in 2011), which contributes great risk for the spread of human immunodeficiency virus (HIV)/acquired immunodeficiency syndrome (AIDS) and other sexually transmitted infections (STIs). According to the most recent surveillance data available, the cumulative reported HIV-infected patients in China exceeded 370,000, including 130,000 who had progressed to AIDS. Four major factors, including drug abuse, migrant workers, unprotected and high-risk sexual activity, and the lack of knowledge about HIV/AIDS, have been identified as significant contributors to the HIV/AIDS epidemic among the general population in China.

Heterosexual contact is now considered the most common mode of transmission of HIV infection in China. Shanghai, one of the largest cities in China, had 1294 new HIV infections in 2011, 82.5% of which were acquired through sexual contact. Eight hundred eighty of the 1294 infections (68.0%) involved rural-to-urban migrant workers. Currently, there are approximately 200 million migrant workers in China, most of whom originated from poorer regions of the country and came to work in the cities as laborers, restaurant workers, and sex workers. The itinerant population is considered as the 'tipping point' for the current HIV/STI epidemic [1,2]. It has been suggested that rural-to-urban migration may play a crucial role in shifting the HIV/AIDS epidemic by broadening social and sexual mixing [3,4]. In many cases, female sex workers (FSWs) from the itinerant population are considered a significant contributor to the heterosexual transmission rates of HIV/STIs because their unprotected, anonymous sexual activities act as a "bridge" to spread HIV/STIs to the general population [5]. Most FSWs in large cities migrate from poor rural areas [6], and are mainly young girls with limited education who are simply unaware of the risk of HIV/STIs. A poor awareness of self-protection is an obstacle to the consistent and proper use of condoms during a commercial transaction. In China, providing commercial sexual services is illegal and most FSWs operate in karaoke bars, massage parlors, saunas, and hair and beauty salons, while some FSWs solicit clients from the street or in parks [7].The prevalence of HIV in FSWs illustrate the critical need for preventive efforts and health education.

HIV/AIDS prevention has been influenced by cognitive theories of behavioral change that emphasize promoting knowledge, perceptions, beliefs, and skills regarding HIV/AIDS [8]. Many studies had focused on HIV/STI prevention among FSWs by health education and condom promotion at the individual level. In recent years, health promotion theories of behavioral change have focused not only on the individual , social and psychological predisposing factors, but also enabling and reinforcing factors, such as environmental-structural factors [9,10]. The most well-known environmental-structural intervention of HIV/AIDS research is the Thai 100% condom program, which requires condom use in all brothel-based commercial sex acts sponsored by the government [11]. Ang and Morisky [12] reported that STI risk among female bar entertainers in the southern Philippines could be reduced by combining manager and peer interventions. Other researchers have concluded that structural and environmental influences, such as the physical, social, policy, and economic environments, are barriers to condom negotiation [13,14]. Urada [15] suggested that interventions should consider the effects of environmental-structural factors on consistent condom use (CCU). The India experience showed a 2.5 times the odds ratio of CCU among those FSWs who were involved in program exposure and high levels of collective agency [16].

The initial success of environmental-structural intervention has inspired related intervention research in other countries, and recently in some pilot regions in China. These interventions are difficult to conduct without the support of FSWs, establishment owners, government and establishment-based policies, and support systems. Because commercial sexual service is illegal in China, investigation and research of FSWs is a sensitive issue which is not easy to conduct, thus limiting related studies in Shanghai. The purpose of the current study was to obtain information about social and psychological and environmental-structural factors in determining CCU among rural-to-urban FSW migrants in five districts of Shanghai using cluster randomized sampling.

## Methods

This study was approved by the Shanghai Medical Ethics Committee and the Shanghai Municipal Health Bureau. All of the participants were informed of the study objectives and provided consent.

### Sample and selection criteria

Shanghai is one of the largest metropolitan areas in China, with a 40% itinerant population in whom HIV/AIDS transmission is largely attributed to heterosexual contact. Because provision of commercial sexual services is illegal in China, and most FSWs generally operate covertly, it is impossible to estimate the actual number of FSWs operating in Shanghai. Non-governmental organizations and community hospitals are the best links to the FSW population, and we relied on their knowledge to conduct our cross-sectional survey by cluster sampling. Between July and December 2009, 20 geographic sites among 5 districts were identified randomly where access to FSWs was considered feasible. One hundred thirty-two small sex establishments, including Xitou Fang, massage parlors, and hair salons from 20 geographic sites of 5 districts which explicitly provided sexual services, were enrolled in the study. We visited every hotspot that provided commercial sexual services and interviewed the FSWs working there. Currently, there are only 4–6 FSWs working in each hotspot. The participant selection criteria included FSW migrants from the mainland who reported that at least one of their last three sexual partners had paid to go out on a date.

### Sample estimation

By conducting a detailed review of the literature and performing a small scale pre-survey with a limited portion of the FSWs of the itinerant population in Shanghai, we found that risky behaviors, such as unprotected sexual intercourse, were practiced by 34%-40% of FSWs [17]. By taking a cross-sectional sample size estimation approach, the error was calculated as follows: *ϵ* = 0.15*P*; P = 37%; Q = 1-P =63%; and *α* = 0.05 *Z*_*α*_ = 1.96. The sample size calculation method used was as follows:

(1) n=zα/22 P1−pϵ2=178(1−p)p=178(1−0.37)0.37=303

The intra-cluster correlation coefficient (ICC) of the primary outcome is known to play a key role in the design and analysis of cluster randomized trials, in which clusters, such as health care organizations, school classes, or geographic areas, are randomized to trial arms and outcomes are measured for individuals within those clusters [18]. The ICC for condom use ranged from 0.01-0.08 in other studies [19,20]. Because it is very difficult to obtain a sufficiently large sample population of FSWs in China, we used the average ICC (0.045) to adjust the sample size, as follows: m = N/layers = 303/20 = 15, where

(2) $$N_{\mathrm{adjueted}}=n\times [1+ICC(m-1)]=303\times [1+0.045(15-1)]=494$$

Our final sample population was comprised of 504 FSWs from the Shanghai district hotpots located in 20 geographic sites.

### Data collection

Researchers from the School of Public health of Shanghai Jiaotong University surveyed the FSWs of small sex establishments using structured questionnaires between May 2010 and January 2011. Individuals completed the questionnaires by interview-led surveys to collect detailed information about condom use and specific knowledge, perceptions, attitudes, behaviors, and the physical, social, and policy environment related to condom use and HIV/AIDS prevention. Prior to participation, we explained the aim and major content of the survey to each of the FSWs and emphasized that participation was voluntary and anonymous. The procedure lasted approximately 30 minutes and was conducted face-to-face in a private room. Each participant was compensated 50 RMB ($7-8 USD) in cash.

### Measure

The questionnaire was developed by referring to the entertainment guide to HIV/AIDS prevention in the China and World Health Organization guidelines. A preliminary research showed good fitness of the questionnaire by reliability and validity analyses. The questionnaire consisted of socio-demographic characteristics of the participants (the age, hometown, year school was completed, civil status, monthly income, age of sexual debut, age at which the participant started selling sex, duration of sex work, and average client dates per week),predisposing factors of condom use (perception of susceptibility to HIV/AIDS , perception of benefits of condom use, and safe sex self-efficacy), and enabling and reinforcing factors, which are usually environmental-structural factors. The method by which we scored the constructs of perception of susceptibility, condom use, and safe sex self-efficacy was based on the Thurstone scale [21].

The perception of susceptibility to HIV/AIDS and benefits of condom use were used to measure the individual perception of susceptibility to HIV/AIDS as follows: “everyone can be infected by HIV;” “HIV-infected people and ordinary people are difficult to separate;” “HIV can be spread through sexual transmission;” and “HIV also can be transmitted by oral sex.”. Correct answers were credited with a score of one, while incorrect answers or responses of “do not know” received a score of zero. The sum of the score of each question was converted into a total score, with a maximum of 4 (Cronbach’s alpha, 0.81). Another four items related to condom use during sexual intercourse were also used to measure the perception of benefits of condom use by the participants, and the sum of the score of each question was converted into a total score, with a maximum of 4 (Cronbach’s alpha, 0.75).

The safe sex self-efficacy was investigated as follows: CCU with clients when sexually excited; reject clients who looked clean and offered more money not to use condoms; reject clients who refused to used a condom; use condoms with intoxicated clients. The sum of the score of each question was converted into a total score, with the maximum of 4 (Cronbach’s alpha, 0.63).

Environmental-structural support was enabling and reinforcing factors supporting condom use in the establishment as follows: the perceived level of safe sex information exchange among employees; support from the establishment owner about the important of condom use during commercial sexual services; accessibility of condoms in the establishment; accessibility of HIV voluntary testing performed by government hospitals or the CDC; support from community physicians about health advise; health education and health promotion on HIV/AIDS from regular intervention for high-risk population; and getting free condom from family planning departments ( Cronbach’s alpha, 0.71).

Consistent condom use was measured using a five-point Likert scale, as follows: “Have you always, almost always, sometimes, almost never or never use condoms during commercial sexual service (the primary dependent variable of our study).” FSWs were considered to use condoms consistently if they answered “always” compared to inconsistently, which was defined as “almost always, sometimes, almost never and never” [22].

### Data entry and analysis

Data were double-entered using Epidata 3.0 software. All statistical analyses were performed using the Statistical Package for Social Sciences (SPSS) for Windows. Generalized assessments were made using the mean, standard deviation, and percentages. A chi-square test was used to compare differences in socio-demographic characteristics between the suburbs and central city. For multivariate analysis, the continuous independent variables were examined for normalcy and were categorized by the median. The dependent variable (condom use) was dichotomized into consistent (always) versus non-consistent (almost always, sometimes, almost never, or never) and a binary logistic regression was used to determine the predictors of CCU. To address potential collinearity among the variables, we added the variables to the regression model at a time by a forward stepwise approach, and the significant level was 0.05.

## Results

### Characteristics of the individual, social, psychological and environmental-structural factors of the sample

The 504 participants who worked in small sex establishments were all rural-to-urban migrants from smaller cities outside of Shanghai. As described in Table 1, most of the FSWs in the survey were young women between 16 and 35 years of age (median age, 25 years), with a low level of formal education (completed 9 years of school on average). Three hundred six FSWs were employed in establishments in the suburbs and 198 FSWs worked downtown. The median monthly income of the FSWs was 2000 RMB (approximately US$310). The median age at the time of sexual debut was 19 years. The average age at which the FSWs began selling sex was 22 years, and women reported working as sex workers for a median of 3 years. The median number of dates with clients per week was 6 among FSWs, and 63.6% of the FSWs reported using condoms consistently with paying partners. The FSWs scored high on the individual safe sex self-efficacy, perception of susceptibility to HIV/AIDS, and benefits of condom use during sexual intercourse (median, 3.0 on a4-item scale). The perceived and observed environmental-structural support for condom use in small sex establishments was low (median, 3.0 on a 7 -item scale).

**Table 1:** Characteristics of individual, social, psychological, and environmental-structural factors of FSWs in Shanghai, China (N = 504)

**Variable**	**Median (range) or count (percentage) in each category**	
Age (years)	25	(16.0-35.0)
Year of school completed	9	( 0–12 )
Duration of sex work (years)	3	(0–8)
Monthly income (RMB [yuan])	2000	(800–8000)
Age of sexual debut	19	(14–27)
Age started selling sex	22	(15–31)
Average client dates per week	6	(2–14)
Civil status:		
Single	299	(59.3)
Married/co-habiting	195	(40.7)
Establishment location:		
Downtown	198	(39.3)
Suburbs	306	(60.7)
Perception of susceptibility on HIV/AIDS	3	(0–4)
Perception of benefits on condom use	3	(0–4)
Safe sex self-efficacy	3	(0–4)
Environment-structural support	3	(0–7)
Consistent condom use:		
Always	319	(63.3)
Less than always	186	(36.7)

### Association between individual, social, psychological, environmental-structural factors and CCU

As shown in Table 2, the relationship between CCU and each individual, social , psychological or environmental-structural support factor was examined first. Age and location of the establishment were the two demographic variables significantly associated with CCU. Young women (16–25 years) were more likely than older women to use condoms consistently with paying partners. Participants employed in downtown establishments were significantly more likely to use condoms consistently with clients than FSWs in the suburbs. The age at which the FSW started selling sex (< 23 years), and the average number of dates with clients per week (> 6) were also significantly associated with high rates of CCU. All four of the aggregate measures developed for the research, including safe sex self-efficacy, perception of susceptibility to HIV/AIDS, perception of benefits of condom use, and environmental-structural support for condom use, were significantly associated with CCU. The rate of reported CCU among these women with high safe sex self-efficacy was 76.6% versus 49.2% among those FSWs with low self- efficacy on safe sex (odds ratio [OR], 3.37; 95% confidence interval [CI], 2.30-4.94].The rate of CCU among FSWs with a high perception of susceptibility to HIV/AIDS (77.9%) was significantly higher than the FSWs with a low perception (53.7%; OR, 3.05; CI, 2.03-4.57). FSWs with a higher perception of benefits of condom use were more likely to use condoms consistently with paying partners than FSWs with a lower perception (76.6% vs. 49.2%; OR, 2.61; CI, 1.75-3.88). The rate of CCU varied significantly between FSWs employed in establishments with high levels of environment-structural support for condom use during sex trade (82.1%) compared with FSWs with low levels (49.8%; OR, 4.63; CI, 3.03-7.06).

**Table 2:** Association between individual, social, psychological, environmental-structural factors and consistent condom use among FSWs in Shanghai, China

**Variable**	**CCU**			**P**	**Unadjusted OR**	**95% CI**
	**N**	**%**	** *χ* **^**2**^			
Year of age						
16-25	188	68.4	7.21	0.007	1.64**	1.14-2.37
26+	130	56.8				
Year of school completed						
0-9	243	61.1	3.38	0.066	1.54	0.97-2.46
10+	75	70.8				
Monthly income (RMB [yuan])						
800-2000	156	68.8	3.37	0.066	1.41	0.98-2.03
2001+	162	57.0				
Establishment location						
Downtown	137	59.3	4.67	0.031	1.51*	1.04-2.21
Suburbs	181	68.8				
Civil status						
Single	208	64.0	0.32	0.571	0.90	0.62-1.31
Married/co-habiting	110	61.5				
Duration of sex work (years)						
0-3	216	65.7	2.66	0.103	1.37	0.94-1.99
4+	102	58.3				
Age of sexual debut						
14-19	201	65.5	2.03	0.154	1.31	0.90-1.89
20+	116	59.2				
Age started selling sex						
16-22	178	68.5	6.64	0.010	1.61*	1.12-2.32
23+	140	57.4				
Average client dates per week						
2-6	166	59.3	4.50	0.034	1.49*	1.03-2.16
7+	152	68.5				
Perception of susceptibility on HIV/AIDS						
Low (0–3)	166	53.7	30.14	<0.001	3.05***	2.03-4.57
High (4)	152	77.9				
Perception of benefits on condom use						
Low (0–3)	169	54.9	23.01	<0.001	2.61***	1.75-3.88
High (4)	149	76.0				
Safe sex self-efficacy						
Low (0–3)	122	49.2	40.52	<0.001	3.37***	2.30-4.94
High (4)	196	76.6				
Environment-structural support						
Low (0–3)	148	49.8	54.64	<0.001	4.63***	3.03-7.06
High (4–7)	170	82.1				

*CCU* consistent condom use, *CI* confidence interval, *OR* odds ratio.

P value: * <0.05; ** <0.01; *** <0.001.

### Determinants of CCU by a multiple logistic regression model

We entered the individual, social, psychological and environmental-structural factors in the multiple logistic regression model using a forward stepwise approach. As shown in Table 3, the final regression model was statistically significant with the following five variables determining the CCU: location of the establishment; safe sex self-efficacy; perception of susceptibility to HIV/AIDS; perception of benefits of condom use; and environmental-structural support for condom use. FSWs with high environmental-structural support for condom use were significantly more likely to use condoms consistently with paying partners than with low environmental-structural support (OR, 3.96; CI, 2.52-6.22). In addition, participants were more likely to use condoms consistently with paying partners if they were employed in downtown establishments (OR, 1.71; CI, 1.12-2.61), had a high perception of susceptibility to HIV/AIDS (OR, 1.96; CI, 1.25-3.01), a high perception of benefits of condom use (OR, 2.06; CI, 1.32-3.22), and perceived a strong sense of self-efficacy on safe sex during commercial sexual services (OR, 2.52; CI, 1.64-3.85).

**Table 3:** Determinants of consistent condom use among FSWs in Shanghai, China using multivariate logistic regression model by a forward stepwise approach (N = 502)

**Variable**	**Adjusted OR**	**95% CI**
Establishment location: downtown	1.70*	1.12-2.61
Perception of susceptibility on HIV/AIDS: high	1.96**	1.25-3.01
Perception of benefits on condom use: high	2.06***	1.32-3.22
Safe sex self-efficacy: high	2.52***	1.44-3.56
Environment-structural support: high	3.96***	2.52-6.22

*CCU* consistent condom use, *CI* confidence interval, *OR* odds ratio.

P value: * <0.05; ** <0.01; *** <0.001.

## Discussion

The results of our research among FSWs in Shanghai provide empirical information on HIV/AIDS prevention, which confirm the importance of social and psychological and environmental-structural factors determining CCU among FSWs and their paying partners. Significant predictors of CCU among FSWs in Shanghai existed at not only individual and social and psychological levels (predisposing factors, such as safe sex self-efficacy, perception of susceptibility to HIV/AIDS, and benefits of condom use), but also at the environmental-structural level (enabling and reinforcing factors, such as support from the establishment owner about the importance of condom use during commercial sexual services, accessibility of condoms in the establishment, and support from community doctors about health advise, health education, and health promotion) At the individual-cognitive level, self-efficacy on safe sex played a main role in CCU based on multivariate analysis, which indicates that it is very important to enhance safe sex behaviors by improving skills, such as how to negotiate safe commercial sex and persuade clients into using condoms consistently during sexual intercourse. Individual perception of susceptibility to HIV/AIDS and benefits of condom use also determined CCU among FSWs, and cognitive information can be easily delivered to FSWs via health education. Last but not least, environmental-structural factors determined CCU definitively among FSWs as evidenced from the multivariate stepwise logistic regression model. The results of the current study were similar to Moriskey [23] in 2011, which indicated that CCU in the Philippines and Hong Kong was significantly associated with interpersonal and venue-level factors. Individuals who had higher appointment-keeping ratios in the Philippines had higher rates of CCU and significantly lower rates of STIs. Moriskey [23] suggested that China develop city ordinances and establishment regulations for regular examinations among FSWs and entertainers in the local STI clinics. Interventions should seek not only to conduct health education at the individual level, but also to promote institutional and community-based solidarity among FSWs, establishment owner/managers, community doctors, family planning departments, and other health service organizations to increase condom use in HIV/STI prevention. The research presented herein will aid in future studies on intervention techniques among FSWs in Shanghai because interventions targeted at FSWs are among the most cost-effective public health strategies available to curb the transmission of HIV.

The commercial sex trade has a long and complex history in China, and has been greatly influenced by the political and economic changes experienced by the Chinese Republic during the 20th century [5,24]. During the past 33 years, the open door policy and economic reforms in China have been accompanied by a remarkable resurgence in the commercial sex sector. Prostitution and extramarital sex is an indisputable fact involved in all social classes. Indeed, the Chinese National Sentinel Surveillance System concluded that sexual transmission was the most common route of new HIV infections between 2007 and 2010. FSWs represent an important reservoir of STIs, including HIV. Providing commercial sexual services is illegal in China and most FSWs operate underground, and in many cases, these FSWs are simply unaware of the risk of HIV/AIDS because most of them are young girls with limited education who have migrated from poor rural areas to towns or cities. Thus, it is more difficult to perform health promotion on HIV/AIDS among high-risk populations, like FSWs.

In July 2004, six national ministries of China implemented a condom promotion program to reduce HIV/AIDS transmission among high-risk populations (entertainment), which reflect the support of the government on a "harm reduction strategy" in HIV/AIDS prevention. Others believe that it is harmful to promote condom use by the government, which appears to encourage the commercial sex trade and make a conflict with the traditional strategies of fighting against prostitution. The contradictions caused by social values conflict in China, in which the government has become involved in the dilemma of HIV/AIDS prevention among FSWs. The prevalence of STIs and HIV in FSWs suggests a critical need for preventive efforts and health education. We suggest that non-governmental organizations (NGOs), universities, and community health service centers should play the main roles in HIV/AIDS prevention among FSWs, and the government should play a role in creating necessary policy and legal conditions to enhance environmental-structural support for condom use instead of conducting interventions directly.

## Conclusions

The findings in this research show that environmental-structural factors support for condom use are significant predictors of CCU among FSWs; however, cross-sectional research design limits our current study to establish causal associations between social, physical, policy, and environmental support and condom use. In corollary research, we will conduct a prospective study in which FSWs will be followed over time, and assess whether or not their sexual behavior will change when they move between small sex establishments that vary in term of levels of environment-structural supports.

## Abbreviations

FSWs: Female sex workers ; CCU: Consistent condom use; HIV/AIDS: Human immunodeficiency virus/Acquired immunodeficiency syndrome; STIs: Sexually transmitted infections; NGOs: Non-governmental organizations.

## Competing interests

The authors declare that they have no competing interests.

## Authors’ contributions

All authors contributed to the design of this research. XXY drafted the manuscript and was involved in the interpretation of the data. YC and MLS performed statistical analyses; TS, BP, and XJ played a major role in the field survey. YC made a substantial contribution to the interpretation of the data and was involved in revision of the manuscript through all stages. All authors read and approved the final manuscript.

## Pre-publication history

The pre-publication history for this paper can be accessed here:

http://www.biomedcentral.com/1471-2458/12/599/prepub

## References

[B1] Hong Y Stanton B Li X Rural-to-urban migrants and the HIV epidemic in China AIDS Behav 2006 10 421 430 10.1007/s10461-005-9039-5 16421651 PMC1791012

[B2] Anderson AF Qingsi Z Hua X Jianfeng B China's floating population and the potential for HIV transmission: a socialbehavioural perspective AIDS Care 2003 15 177 185 10.1080/0954012031000068326 12856339

[B3] Hu Z Liu H Li X Stanton B Chen X HIV-related sexual behaviour among migrants and non-migrants in a rural area of China: role of rural-to-urban migration Public Health 2006 120 339 345 10.1016/j.puhe.2005.10.016 16473377

[B4] He N Detels R Chen Z Jiang Q Zhu J Dai Y Wu M Zhong X Fu C Gui D Sexual behavior among employed male rural migrants in Shanghai, China AIDS Educ Prev 2006 18 176 186 10.1521/aeap.2006.18.2.176 16649962

[B5] Yingying H Gaile H Suiming P Myrons C HIV/AIDS risk among brothel-based female sex workers in China: Assessing the terms, content, and knowledge of sex work Sex Transm Dis 2004 31 11 695 700 10.1097/01.olq.0000143107.06988.ea 15502679

[B6] Rou Keming W Zunyou SSG A five-city trial of a behavioural intervention to reduce sexually transmitted disease/HIV risk among sex workers in China AIDS 2007 21 suppl 8 S95 S101 10.1097/01.aids.0000304703.77755.c7 18172399

[B7] Hershatter G Dangerous Pleasure. Prostitution and Modernity in Twentieth-Century Shanghai 1997 Berkeley: University of California Press

[B8] Kelly JA Muphy DA Psychological interventions with AIDS and HIV: prevention and treatment J Consult Clin Psychol 1992 60 576 585 1506505 10.1037//0022-006x.60.4.576

[B9] Kerrigan D Ellen JM Moreno L Environmental-structural factors significantly associated with consistent condom use among female sex workers in the Dominican Republic AIDS 2003 17 415 423 10.1097/00002030-200302140-00016 12556696

[B10] Sumartojo E Structural factors and HIV prevention AIDS 2000 14 Suppl.1 S22 S23 10981469 10.1097/00002030-200006001-00002

[B11] Rojanapithayakorn W Hanenberg R The 100% condom program in Thailand AIDS 1996 10 1 7 8924236 10.1097/00002030-199601000-00001

[B12] Ang A Morisky DE A mutilevel analysis of the impact of socio-structural and environmental influence on condom use among female sex works AIDS Behv 2012 16 934 942 10.1007/s10461-011-9925-y PMC3165094 21431414

[B13] Shannon K Structural and environmental barriers to condom use negotiation with clients among female sex workers: implications for HIV-prevention strategies and policy Am J Public Health 2009 99 4 659 665 10.2105/AJPH.2007.129858 19197086 PMC2661482

[B14] Urada LA Condom negotiations among female sex workers in the Philippines: environmental influences PLoS One 2012 7 3 e33282 10.1371/journal.pone.0033282 22448241 PMC3308968

[B15] Urada L Morisky D Hernandez L Strathdee SA Social and structural factors associated with consistent condom use among female entertainment workers trading sex in the Philippines AIDS and Behavior 2012 in press 10.1007/s10461-011-0113-x PMC3987851 22223297

[B16] Blankenship KM Power, community mobilization, and condom use practices among female sex workers in Andhra Pradesh, India AIDS 2008 22 Suppl 5 S109 S116 10.1097/01.aids.0000343769.92949.dd 19098471

[B17] Yong C Rong S Tian S Bei P Xueqin J Xiuxia Y Gang X Shenghui L Hong H Meili S A study of HIV/AIDS related knowledge, attitude and behaviors among female sex workers in Shanghai China BMC Publ Health 2010 10 377 10.1186/1471-2458-10-377 PMC2908579 20584296

[B18] Eldridge SM Ukoumunne OC Carlin JB The intra-cluster correlation coefficient in cluster randomized trials: a review of definitions Int Stat Rev 2009 77 3 378 394 10.1111/j.1751-5823.2009.00092.x

[B19] Feldblum PJ Kuyoh M Omari M Ryan KA Bwayo JJ Welsh M Baseline STD prevalence in a community intervention trial of the female condom in Kenya Sex Transm Inf 2000 76 454 456 10.1136/sti.76.6.454 PMC1744233 11221128

[B20] Feldblum Paul J Kuyoh Maureen A Bwayo Job J Omari M Wong Emelita L Tweedy Kathryn G Welsh Michael J Female condom introduction and sexually transmitted infection prevalence: results of a community intervention trial in Kenya AIDS 2001 15 8 1037 1044 10.1097/00002030-200105250-00012 11399986

[B21] Krabbe PF Thurstone scaling as a measurement method to quantify subjective health outcomes Med Care 2008 46 4 357 365 10.1097/MLR.0b013e31815ceca9 18362814

[B22] Deering KN Paranita B Janet B Condom use within non-commercial parnerships of female sex workers in southern India BMC Publ Health 2011 11 Suppl 6 S11 10.1186/1471-2458-11-S6-S11 PMC3287549 22376171

[B23] Morisky DE Urada LA Organizational policy recommendations for control of STI/HIV among female sex workers in China: regular examination of workers in social hygiene clinics AIDS Care 2011 23 Suppl 1 83 95 21660754 10.1080/09540121.2011.554520 PMC3117269

[B24] Shan GN Zhongguo Changji: Guoqu yu Xianzai (Prostitutes in China:The Past and Present) 1995 Beijing: Falu Chubanshe Press

